# New Insights of CCR7 Signaling in Dendritic Cell Migration and Inflammatory Diseases

**DOI:** 10.3389/fphar.2022.841687

**Published:** 2022-02-25

**Authors:** Wenxiang Hong, Bo Yang, Qiaojun He, Jiajia Wang, Qinjie Weng

**Affiliations:** ^1^ Center for Drug Safety Evaluation and Research, Zhejiang Province Key Laboratory of Anti-Cancer Drug Research, College of Pharmaceutical Sciences, Zhejiang University, Hangzhou, China; ^2^ The Second Affiliated Hospital, Zhejiang University School of Medicine, Hangzhou, China; ^3^ Westlake Laboratory of Life Sciences and Biomedicine, Hangzhou, China

**Keywords:** CCR7, dendritic cells, migration, regulatory network, immunotherapy

## Abstract

CCR7, collaborated with its ligands CCL19 and CCL21, controls extensive migratory events in the immune system. CCR7-bearing dendritic cells can swarm into T-cell zones in lymph nodes, initiating the antigen presentation and T-cell response. Abnormal expression of CCR7 in dendritic cells will cause a series of inflammatory diseases due to the chaotic dendritic cell trafficking. In this review, we take an in-depth look at the structural–functional domains of CCR7 and CCR7-bearing dendritic cell trajectory to lymph nodes. Then, we summarize the regulatory network of CCR7, including transcriptional regulation, translational and posttranslational regulation, internalization, desensitization, and recycling. Furthermore, the potential strategies of targeting the CCR7 network to regulate dendritic cell migration and to deal with inflammatory diseases are integrated, which not only emphasizes the possibility of CCR7 to be a potential target of immunotherapy but also has an implication on the homing of dendritic cells to benefit inflammatory diseases.

## Introduction

Dendritic cells (DCs) are the center of both innate and acquired immunity, embodied in their role in the recognition of internal and external antigens and immunomodulators (Banchereau and Steinman, 1998; Minton, 2017). DCs mainly incorporate “conventional” or “classical” DCs (cDCs) and plasmacytoid DCs (pDCs) (Worbs et al., 2017; Collin and Bigley, 2018). cDCs are classical antigen-presenting cells, while pDCs are the main producers of type I IFNs, which further serve innate immunity. Owing to their role in immunogenicity and immunological tolerance, they can shape various immune disease courses by differentiating T cells into various subtypes and initiating homologous immunoreaction. However, the migration of DCs, which runs through the life cycle of DCs’ differentiation and development, is the premise for DCs to perform their biological function *in vivo*.

The pre-DCs or immature DCs (iDCs) in blood circulation can migrate to the peripheral tissues, where they can be converted into mature DCs (mDCs) with high expression of MHC molecules and CD80/CD86 costimulator molecules by receptor-mediated endocytosis, phagocytosis, and macropinocytosis of antigens. Subsequently, antigen-carrying mDCs upregulate CC chemokine receptor 7 (CCR7), a 7-fold transmembrane G protein-coupled receptor (GPCR), and are home to draining lymph nodes (dLNs) where they can activate T cells (Teijeira et al., 2014; Worbs et al., 2017). CCR7 mediates not only the migration of cDCs but also the trafficking of pDCs to dLNs (Worbs et al., 2017; Mitchell et al., 2018). In addition, under certain conditions, such as the steady state, peripheral DCs can take up self or foreign harmless antigens and move to dLNs continuously, albeit at low frequencies, which may prompt immunological tolerance (Guindi et al., 2018; Falcon-Beas et al., 2019). *Ccr7*-deficient mice greatly verify the idea that the migration of those so-called “semi-mature” DCs or “tolerogenic” DCs to lymph nodes also relies on CCR7.

CCR7 has two CC motif ligands, CCL19 and CCL21. The CCR7-CCL19/21 axis has been established as a pivotal component in the trafficking of various immune cells to the lymph nodes and even the metastasis or invasion of some malignant tumor cells (Forster et al., 2008; Mishan et al., 2016). CCR7 undergoes a conformational change after ligand stimulation, which will elicit a series of G protein-dependent or -independent intracellular signaling pathways, such as MEK/MAPK, Rho/cofilin, and PI3K/AKT (Liu et al., 2019; Yoo et al., 2019; Rodriguez-Fernandez and Criado-Garcia, 2020), thus changing Ca^2+^ flux (Crottes et al., 2016), metabolism levels (Liu et al., 2019), and polarity or adhesion of DCs (Joosten et al., 2018) and finally accelerating their chemotactic movement.

Increasing investigations suggest that DC migration is strictly relevant to many immune-related diseases, such as dermatomyositis, atherosclerosis, and viral infections (Lv et al., 2018; Ting Wang et al., 2019; Yan et al., 2019). Using *Ccr7* conditional knockout mice, *Ccr*7^−/−^ DCs, or other intervention methods, we can see that inappropriate regulation of CCR7-mediated DC migration may affect the occurrence, progression, and amelioration of the aforementioned diseases (Comerford et al., 2013).

In this review, we summarized the various functional domains of CCR7 and the trajectory of CCR7-CCL19/21 axis-mediated DC migration. Particularly, we focus on the regulatory network of CCR7 signaling, exploring feasible strategies to weaken or reinforce DC migration *via* targeting the CCR7 network to benefit clinical therapy, highlighting the potential of CCR7 to become an effective intervention target.

## Overview of the CCR7-CCL19/CCL21 Axis

### CCR7 and Its Ligands

**FIGURE 1:**
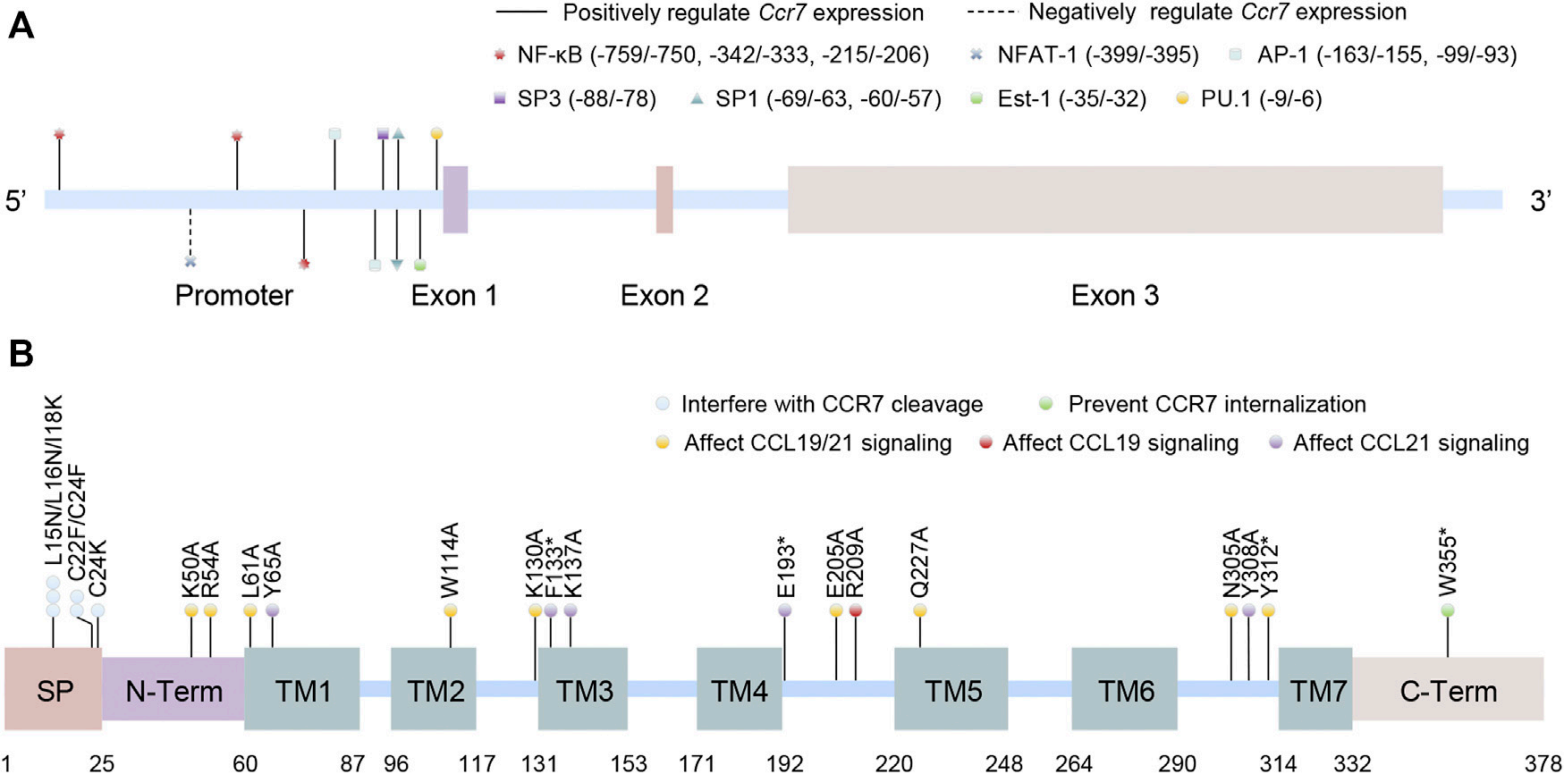
Schematic structures of the human CCR7 gene and protein. **(A)** The location of promoter and exons of *Ccr7* as well as the binding sites of transcription factors are shown in the *Ccr7* gene (the first base of exon 1 is set to +1). **(B)** The protein domains of CCR7 are presented. Artificial mutations in different domains or locus are contacted to the function of CCR7. SP: cleavable signal peptide; N-Term: N-terminal extracellular domain; TM: transmembrane spanning alpha helices domain; and C-Term: C-terminal cytoplasmic domain.

CCR7, encoded by the *Ccr7* gene located in chromosome 17q12-q21.2 in humans (including three exon sequences), is the first identified lymphocyte-specific GPCR (Figure 1A)(Birkenbach et al., 1993). Although no variant of the *Ccr7* gene is found to be directly linked to diseases in humans, it is claimed that the *Ccr7* variant could potentially lead to increased susceptibility to autoimmunity (Kahlmann et al., 2007). The CCR7 protein consists of 378 amino acids and can be divided into an N-terminal extracellular domain, a C-terminal cytoplasmic domain, and seven transmembrane spanning alpha helices (TM1–TM7) like other chemokine receptors. However, it possesses a cleavable 24 amino acid-long signal peptide that is unique among chemokine receptors (Figure 1B). Mutations in different domains will bring about biased effects. The unique cleavable signal sequence promotes efficient endoplasmic reticulum to Golgi trafficking of CCR7, mutations of which within the central hydrophobic h-region or the short polar c-region would interfere with its cleavage and CCR7 surface expression (Uetz-von Allmen et al., 2018). Otherwise, the N-terminal extracellular domain, the TM1–7 domains, and the extracellular loop domains are significant for ligand binding and efficacy, probably because of the formation of major and minor binding pockets of chemokines. Some mutations in these domains fine-tune CCL19 but not CCL21 signaling, while some inversely and others both (Ott et al., 2004; Hjorto et al., 2016; Jorgensen et al., 2019). Finally, there are many mutations in the C-terminal cytoplasmic domain of CCR7 relevant to adult T-cell leukemia/lymphoma, and mutation at the Trp355* residue was shown to prevent CCR7 internalization, resulting in increased surface receptor expression and a gain of chemotaxis function (Kataoka et al., 2015).

Chemokines (comprising four major subfamilies: CXC, CC, CX3C, and XC) are soluble small-molecule cytokines or secreted signaling proteins with molecular weights of 8–12 kD. Nearly twenty signaling chemokine receptors have been identified to date, and over fifty chemokine ligands are presented in humans (Comerford et al., 2013). The sole ligands for CCR7 are CCL19 (also known as MIP-3β, Exodus-3, or CKβ11) and CCL21 (SLC, thymus-derived chemotactic agent 4, or Exodus-2), both of which have a conserved tetra-cysteine motif. The main difference between them is that CCL21 has a unique C-terminal tail extension covering an extra 37 amino acids (including six cysteine residues), allowing its combination to glycosaminoglycans or certain components of the extracellular matrix. In contrast, CCL19 does not own this property and is always present in a soluble form (Jorgensen et al., 2018). Besides, multiple investigations have shown that they also differ in magnitude, intensity, and duration of function together with biased signaling pathways (Hjorto et al., 2016; Zhongnan Wang et al., 2019).

### Trajectory of mDC Migration to Lymph Nodes

**FIGURE 2:**
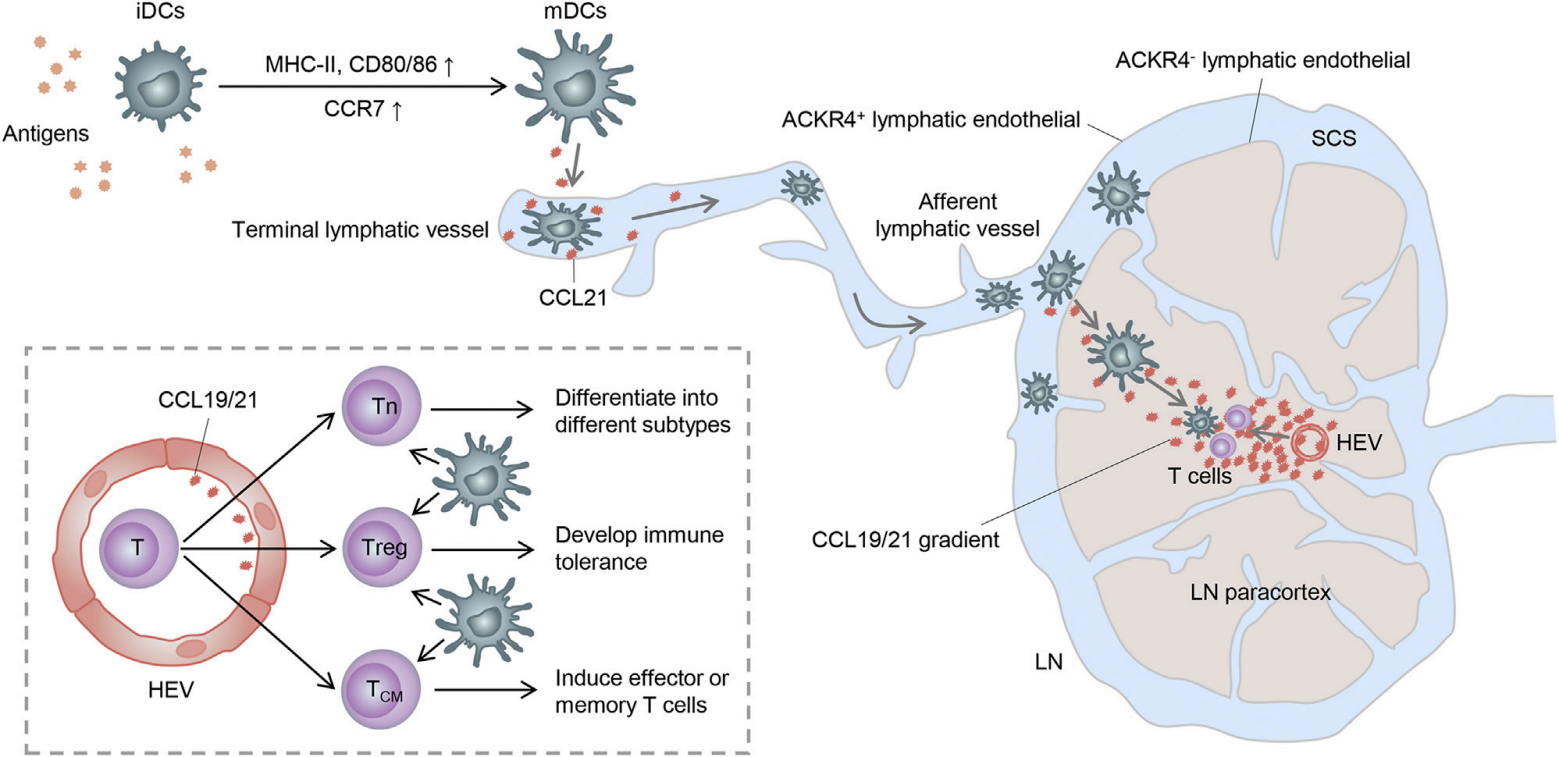
Trajectory of mDC migration to lymph nodes. Stimulated by antigens, iDCs become mDCs and upregulate CCR7, enabling it to enter the lymphatic vessels by the interaction with the CCR7 ligand released here. mDCs then migrate to the SCS and receive a further chemokine gradient pointing toward the T-cell zone. Finally, mDCs get to the lymph node paracortex and activate T cells to complete immunogenicity or tolerance induction. Tn: naive T cell; Treg: regulatory T cell; and TCM: central memory T cell.

As described before, DCs can successfully swarm into T-cell zones of dLNs *via* the CCR7-CCL19/CCL21 axis. However, how do they get into the lymph nodes and how do they locate to the T-cell zones correctly? In this section, we present the main processes of CCR7-mediated migration and localization of DCs in dLNs, along with the subsequent contact and activation of T cells (Figure 2).

It is known that CCR7 can be expressed by various subsets of immune cells, such as DCs, T cells, and B cells, as well as some tumor cells, acting as a key adjustor in guiding them to destinations. Both CCL19 and CCL21 are predominantly secreted by the high endothelial venules (HEVs) and reticular stromal cells (fibroblastic reticular cells, FRCs) in the T-cell-rich paracortical regions of dLNs, as well as in the thymus and the spleen (Randolph et al., 2005; Comerford et al., 2013). Specifically, CCL21 can also be expressed by endothelial cells of afferent lymphatic vessels, whereas CCL19 can be expressed by the migratory DCs in the luminal side of HEVs, which may facilitate the expanded recruitment of DCs (Steen et al., 2014). Based on these, CCL19 and CCL21 can synergistically induce the directional migration of CCR7-bearing DCs to dLNs.

In non-lymphoid tissues, iDCs become semi-mature DCs spontaneously or differentiate into mDCs under inflammation or “danger signals” (such as toll-like receptor signaling) (Banchereau and Steinman, 1998; Minton, 2017). These DCs upregulate the expression of CCR7 and migrate into lymphatic vessels by interacting with its ligands expressed in the terminal lymphatic vessels (Worbs et al., 2017). Because of the discontinuous basal membrane with button-like tight junctions in the initial segment of lymphatic vessels and the formation of overlapping flaps between endothelial cells, DCs can smoothly enter lymphatic vessels through the openings between these buttons (Baluk et al., 2007). Consequently, DCs carried by the lymphatic flow migrate through the afferent lymphatic vessels into the subcapsular sinus (SCS) of dLNs (Braun et al., 2011). In the SCS, lymphatic endothelial cells (LECs) of the ceiling layer express atypical chemokine receptor 4 (ACKR4) and clear the chemokine, thus creating a further chemokine gradient pointing toward the T-cell-rich paracortex (Bryce et al., 2016). Moreover, ACKR2, also expressed on LECs, can withdraw inappropriate inflammatory chemokines attached to LECs and heighten the selective presentation of CCR7 ligands to mDCs (McKimmie et al., 2013). Within the paracortex, DCs can migrate along the FRC networks and the established chemokine gradient under the guidance of CCL21 and CCL19 expressed by FRCs and HEVs (Braun et al., 2011). Importantly, T cells can also enter the lymph node parenchyma from the circulation system *via* HEVs and then have an apparently random “walk” pattern of motion on reticular stromal cells, which may also contribute to them encountering DCs and subsequently being activated (Comerford et al., 2013).

In brief, only by the migration in a unique way can DCs finally meet T cells in dLNs and sensitize them into different subgroups with corresponding functions, thereby exerting the immunogenicity or tolerance effect. Although CCR7 also participates in other biological activities of DCs, such as their proliferation, survival, and maturity (Hu et al., 2017; Burgoyne et al., 2019), all of these may finally contribute to the core function, that is, steering DCs to dLNs.

## The Regulatory Network to CCR7

**FIGURE 3:**
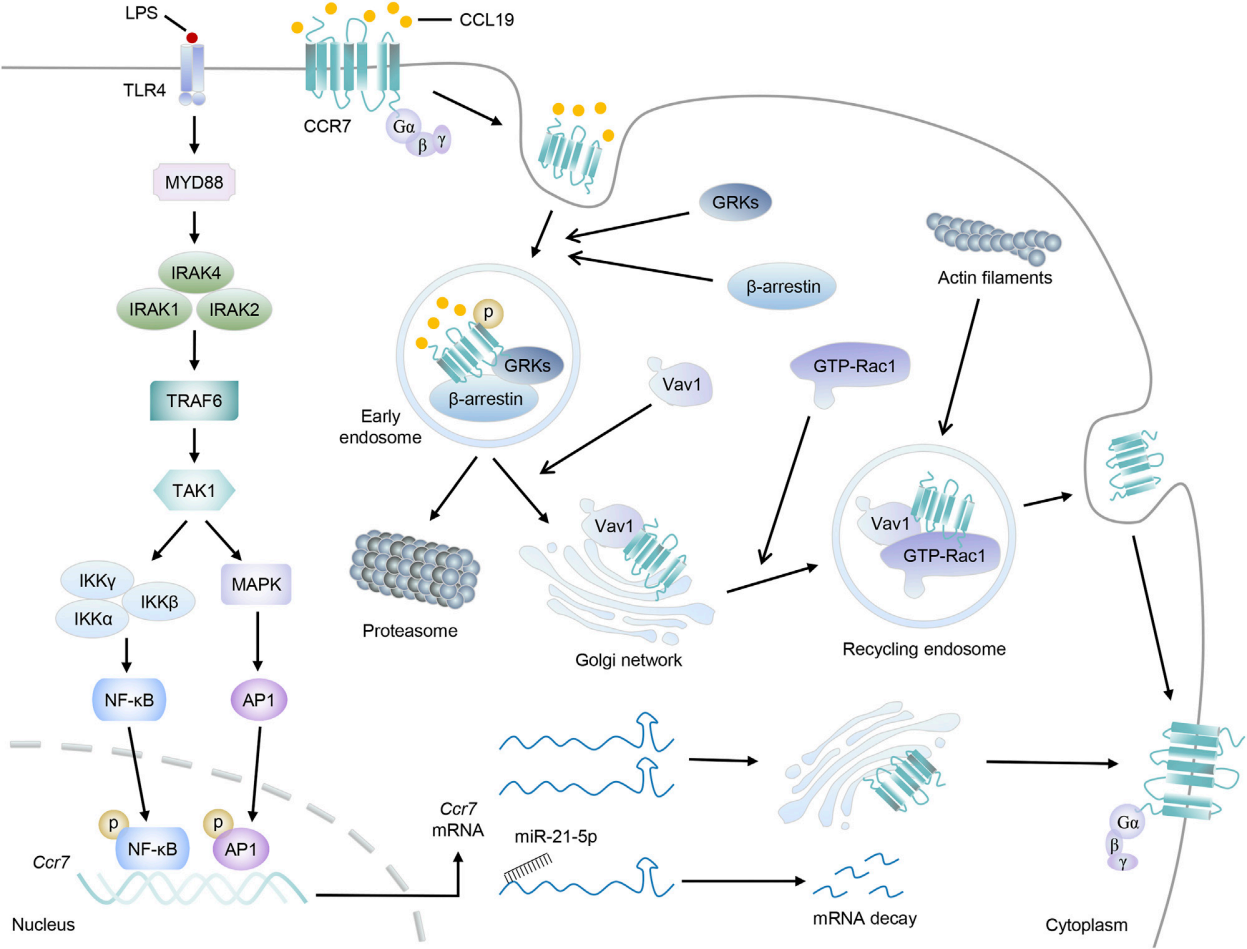
The regulatory network to CCR7. Taking LPS stimulation as an example, the Myd88-TRAF6 signal pathway is activated, which then promotes the transduction of transcription factors NF-κβ and AP-1 to the nucleus to induce the transcription of *Ccr7*. After leaving the nucleus, *Ccr7* mRNAs will locate to the Golgi network for *de novo* synthesis and receptor trafficking of CCR7 protein. Otherwise, *Ccr7* mRNAs interact with miR-21–5p and undergo posttranscriptional regulation for degradation. On the other hand, upon CCL19 stimulation, CCR7 will be phosphorylated by GRKs, accompanied by the recruitment of β-arrestin, and form endosomal vesicles. Then, CCL19 will be sorted to lysosomal degradation while CCR7 can move to the Golgi network, where they will recruit RhoGEF Vav1 and its effector, GTP-bound Rac1. Finally, endomembrane-residing multi-protein-signaling complex comprising CCR7 can recycle back to the plasma membrane and promote the accumulation of actin filaments and cytoskeleton rearrangement.

In view of the paramount importance of CCR7 in DC migration and abundant immunoinflammatory diseases, factors that interfere with the expression or activity of CCR7 signaling are worthy of further research. In this section, we discuss the main mechanisms by which CCR7 is tightly regulated (Figure 3).

### Transcriptional Regulation of CCR7

Evidently, mediating the transcription of *Ccr7* may be the most direct way to affect CCR7 expression (Figure 1). Inspection of the *Ccr7* promoter sequence first identified the binding sites of nuclear factor kappaB (NF-κB) and activator protein-1 (AP-1), both of which can be activated by antigen-induced TLR signaling in DCs (Schweickart et al., 1994; Sun et al., 2018). After receiving low-dose radiation, DC migration and *Ccr7* expression together with the nuclear translocation of p65 (a subunit of NF-κB) were markedly augmented, which may be elucidated by the binding of NF-κB to the *Ccr7* transcriptional promoter and subsequent transcriptional activation (Yu et al., 2018). Although luciferase, ChIP, and EMSA characterized the binding of NF-κB to the promoter region of *Ccr7*, NF-κB inhibition only resulted in partial reduction in CCR7 expression. Further research found the cooperation between NF-κB and AP-1 was crucial to the transcription of *Ccr7* (Mburu et al., 2012; Hong et al., 2016).

Ets-1, a prototype of the ETS family that contains a unique winged helix-turn-helix ETS domain to recognize the GGAA/T core motif in target genes, can bind to the upstream of the transcription start site in the *Ccr7* gene (Fang et al., 2019), and it has also been shown to interact with NF-κB to cooperatively activate *Ccr7* transcription (Mburu et al., 2012; Fang et al., 2019). Moreover, another Ets family protein, hematopoietic lineage-specific transcription factor PU.1, also transactivates the *Ccr7* gene *via* binding to the most proximal Ets motif of the *Ccr7* promoter, thus playing a pivotal role in DC migration (Yashiro et al., 2019). In addition, it was found that IRF4 and IRF8 are required for the development of CD11b^+^ and CD11b^−^ DCs, separately, and are vital for DC migration to lymph nodes during homeostasis and cutaneous inflammation (Schiavoni et al., 2004; Bajana et al., 2012; Vander Lugt et al., 2014). Integration of ChIP-seq and gene-expression analyses indicated that both IRF4 and IRF8 can target and activate *Ccr7* upon DC maturation (Vander Lugt et al., 2014). However, whether they function alone by binding interferon-stimulated response elements or are recruited to composite IRF/PU.1 binding sites on *Ccr7,* or even regulate *Ccr7* indirectly in collaboration with other transcription factors such as NF-κB and AP-1, is still unclear (Marecki and Fenton, 2002; Vander Lugt et al., 2014).

In addition, Sp1 and Sp3 also bind to their potential binding sites in the promoter region of *Ccr7* (Al Akoum et al., 2015). It is well known that pro-inflammatory mediator cyclooxygenase-2 (COX-2) and its metabolite prostaglandin E2 (PGE2) play a profound role in inflammatory responses. Studies also found that COX-2 and PGE2 were able to promote CCR7 expression and DC migration *via* EP2/EP4 receptors (Legler et al., 2006; Pan et al., 2008). Mechanistically, COX-2 and PGE2 increased the phosphorylation of Thr-308 of AKT, which further directly phosphorylate Sp1 at S42, T679, and S698 and enhance the binding of Sp1 to the *Ccr7* promoter (Pan et al., 2008; Chuang et al., 2013).

Previous research has made it clear that FOXO1 can modulate immune response, such as controlling cytokine production and regulating T-cell functions. The direct interaction between FOXO1 and the *Ccr7* promoter in DCs was demonstrated by ChIP and luciferase assay, the intensity of which was elevated after LPS or bacteria stimulation. Based on this, FOXO1 deletion in DCs impaired their homing to dLNs (Dong et al., 2015). However, the exact binding sites of FOXO1 on human *Ccr7* need further research. Moreover, transcription factor NR4A3 is a member of the nuclear receptor *Nr4a* family and highly expressed in migratory DCs. The absence of *Nr4a3* in DCs impaired their migration to dLNs due to the inadequate CCR7 expression. Though no evidence has been found for a direct binding of NR4A3 in the *Ccr7* promoter, *Nr4a3*-deficient DCs had an increased AKT phosphorylation level, which further directed FOXO1 for polyubiquitination and degradation, finally inhibiting FOXO1 activity and DC migration (Park et al., 2016; Yu et al., 2019).

Although transcription factors function by activating transcription in a promoter-specific manner in most cases, they can still work oppositely *via* multifarious ways. Up to now, few transcription factors have been found to repress the transcription of *Ccr7*. Among these, NFAT-1 is best known to bind to the *Ccr7* promoter directly. Other transcription factors such as RUNX3, peroxisome proliferator-activated receptor (PPAR) γ, and liver X receptor (LXR) α also have a certain inhibitory effect on CCR7 expression and DC migration with undefined combination to *Ccr7* (Fainaru et al., 2005; Hanley et al., 2010; Villablanca et al., 2010; Al Akoum et al., 2015). Based on this, research also deemed that PGE2 can upregulate CCR7 at least partly, *via* inhibiting the activation of LXRα in monocyte-derived DCs (moDCs), so as to promote the infiltration of DCs in the tumor site and avoid tumor immune escape (Youlin et al., 2018).

Epigenetic modification, such as histone modification and DNA methylation, is a major event essential for regulating gene expression through chromatin reorganization, thus also involved in the transcription of *Ccr7*. For instance, polycomb repressive complex 2 (PRC2) can be recruited to the specific sites of genome, compacting chromatin and repressing target gene expression through trimethylation of histone 3 lysine 27 (H3K27me3) (Wang et al., 2020). It was found that there was a high level of H3K27me3 on the *Ccr7* locus of lung moDC but not that of migratory cDCs, which may also be closely related to the low expression of CCR7 and the inability of moDC migration (Moran et al., 2014). In line with this, miR-155 directly targets Jarid2, a component of PCR2, and indirectly diminishes the presence of PRC2 and H3K27me3 at the *Ccr7* locus, leading to enhanced expression of CCR7 in DCs (Wang et al., 2016). Likewise, histone deacetylation can also silence gene *via* chromatin compaction. The use of the histone deacetylase inhibitor testified that it can fortify *Ccr7* transcription *via* reversing the promoter hypoacetylation (Mori et al., 2005; Lingling Yang et al., 2018).

Finally, the effect of posttranscriptional regulation, mainly embodied in the splicing of precursor mRNA, the transport from the nucleus to cytoplasm, and the stability of mRNA, is also momentous to the *Ccr7* mRNA. This way, emerging studies have shown that some microRNAs can bind to the 3′untranslational region of *Ccr7* mRNA to regulate its stability and translation. miR-21–5p, which can be enriched in the mesenchymal stromal cell-derived extracellular vesicles, was found to impair the migration ability of DCs by targeting the *Ccr7* mRNA for degradation directly (Reis et al., 2018). Homoplastically, miR-199a-5p, let-7a, and let-7e-5p were also confirmed to be able to target *Ccr7* mRNA and control the metastasis of cancer cells, despite their impact in DC migration being still indistinct (Zhou et al., 2016; Tang et al., 2018; Song Wang et al., 2019).

### Translational and Posttranslational Regulation of CCR7

Except for the aforementioned factors that interfere with the transcription or stability of *Ccr7*, several observations also underline the significance of polysialylation and other posttranslational modifications of CCR7 in DC migration.

Structurally, the most remarkable difference between CCL19 and CCL21 is the unique C-terminal positively charged tail extension of CCL21, permitting its combination to glycosaminoglycans (Jorgensen et al., 2018). Hence, it is tempting to speculate that glycosylation of CCR7 may alter its binding potencies to CCL19 and CCL21. The addition of polysialic acid to N- and/or O-linked glycans, referred to as polysialylation, a rare posttranslational modification executed by ST8Sia II or ST8Sia IV, can also adjust CCR7 activity (Kiermaier et al., 2016). In *St8Sia4*-deficient mice, migration of DCs to dLNs was almost completely abrogated, whereas the migration from SCS to T-cell-rich parenchyma was not affected, which may be explained by the exclusive release of CCL21 but not CCL19 in afferent lymphatic vessels. Additionally, the polySia–CCL21 interaction can promote chemotaxis by releasing CCL21 from an otherwise autoinhibited conformation rather than enhancing CCL21 binding to CCR7 (Kiermaier et al., 2016).

A growing number of research studies have shown that homo- or hetero-oligomerization is essential for CCR7 signaling (Hauser et al., 2016; Kobayashi et al., 2017). Both inflammatory stimuli and PGE2 can induce oligomerization of CCR7, which ulteriorly initiate distinct signaling pathways, enhancing Src activity and CCR7 chemotaxis (Hauser et al., 2016). In addition, phosphorylation and ubiquitination of CCR7 have been attested to be vital for the internalization of CCR7 (discussed in the next section) (Zidar et al., 2009; Schaeuble et al., 2012). Another modification that regulates CCR7-dependent DC migration may be the sulfation of CCR7 N-terminal tyrosine residues. Not only N-terminal phosphotyrosine but also sulfotyrosine-modified CCR7 peptides showed intensive affinity for CCL21, making it possible to become a new would-be drug target (Phillips et al., 2017).

### Internalization, Desensitization, and Recycling of CCR7

It is well known that the β-arrestin-dependent signaling is critical for the internalization, desensitization, recycling, and resensitization of GPCRs (Pavlos and Friedman, 2017). CCL19, but not CCL21, can stimulate this process of CCR7 and lead to the termination of its susceptibility to extracellular ligands (Otero et al., 2006; Hauser and Legler, 2016). Overall, after CCL19 stimulation, activated CCR7 is able to recruit G protein-coupled receptor kinases (GRKs), accompanied by the phosphorylation of CCR7 serine and threonine residues. Afterward, β-arrestin changes from an inactive crystal structure to a high-receptor-affinity structure and then binds to phosphorylated CCR7 to form a GRK/CCR7/β-arrestin trimer (Zidar et al., 2009; Pavlos and Friedman, 2017). The recruitment of GRKs and β-arrestin leads to the formation of endosomal vesicles, which will effectively prevent continuous ligand–receptor nexus. Based on this, some proteins related to endocytosis or vesicle formation, such as clathrin, adapter protein 2, dynamin II, Eps15, and Rab5, were found to be involved in β-arrestin-mediated receptor internalization (Otero et al., 2006; Charest-Morin et al., 2013; Mayberry et al., 2019; Mu et al., 2019).

Endocytosed GPCRs have two outcomes: being sorted into lysosomes for degradation and terminating signal transduction or cycling to an active state and returning to the plasma membrane (Bahouth and Nooh, 2017). It is generally considered that internalized CCL19 will be sorted to lysosomes for degradation, whereas CCR7 can be recycled, although the underlying mechanisms are still poorly understood (Otero et al., 2006). “Fresh” or ligand-free CCR7 can identify chemokines and mediate migration again, which is crucial to ensure continuous directional steering and appropriate responses of DCs. According to a recent study, mDCs possess distinct intracellular pools of CCR7 derived from the plasma membrane or the secretory pathway of newly synthesized receptors, and they always resided at the trans-Golgi network (TGN). At the plasma membrane, Src kinase can phosphorylate the 155-tyrosine residue of CCR7 and then translocate it to endomembranes. Further investigations elucidated that it was required for the recruitment of RhoGEF Vav1 and its effector, Rac1, which will be beneficial for the vesicular trafficking of CCR7, the lamellipodia formation at the cell’s leading edge, and sustained directed migration of DCs (Laufer et al., 2019). Interestingly, CCR7 is ubiquitylated in a constitutive, ligand-independent manner, although its lysineless mutant can be properly inserted into the plasma membrane and had a comparable expression level. Both CCR7 and ubiquitin-defective CCR7-7K7R were internalized normally upon CCL19 binding. However, only wild-type CCR7 can be recycled back toward the plasma membrane *via* TGN, while the CCR7-7K7R mutant was transiently accumulated in the TGN. The lessened CCR7-7K7R-containing globular recycling compartments and the defective migration of DCs additionally strengthened the assumption that ubiquitylation played a role in CCR7 recycling (Schaeuble et al., 2012).

## Potential Strategies to Benefit Inflammatory Diseases *via* Interfering With CCR7 Signaling

Moderate DC migration may be beneficial for immunological stress while excessive CCR7 signal in DCs will lead to an outbreak of inflammation, thus participating in a variety of diseases, such as autoimmune diseases, transplant rejection, and tumor immunology (Seyfizadeh et al., 2016; Brandum et al., 2021). Hence, it is vital to modulate CCR7 signaling tightly to ensure appropriate immunoreaction. Interfering with CCR7 directly or its downstream events holds great promise for CCR7-based immunotherapy. In this section, we focus on the potential strategies to regulate DC migration *via* CCR7 signaling to benefit clinical practice (Table 1).

**TABLE 1:** Potential strategies for immunotherapy *via* affecting the CCR7 system and DC migration.

Strategies/dugs	Mechanism	Models	References
8H3-16A12	Anti-CCR7 Ab	Rheumatoid arthritis	Moschovakis et al. (2019)
CCR7 blocking fusion protein	Block CCR7	Corneal transplantation	Hos et al. (2016)
Anti-TNF Ab	Weaken CCR7 expression	Psoriasis	Bose et al. (2013)
FTY720	Weaken CCR7 expression	Rheumatoid arthritis	Han et al. (2015)
Rosiglitazone and ciglitazone	Activate PPARγ to downregulate CCR7	Lung inflammation	Hammad et al. (2004)
Zaragozic acid	Block cholesterol and oxysterol synthesis to upregulate CCR7	Lymphoma	Villablanca et al. (2010)
M-COSA/OVA/*Ccr7* vaccination	Vaccine targeted DCs to overexpress CCR7	Melanoma	Xiqin Yang et al. (2018)
Thyroid hormone triiodothyronine-treated DCs	DC vaccine with high expression of CCR7	Melanoma	Alamino et al. (2015)
Allogeneic melanoma-derived cell lysate-treated DCs	DC vaccine with high expression of CCR7	Melanoma	Gonzalez et al. (2014)
Protein kinase C inhibitor-processed iDCs	iDC vaccine with high expression of CCR7	Rheumatoid arthritis, primary Sjögren’s syndrome	Adnan et al. (2016)
CCR7-modified iDCs	iDC vaccine with high expression of CCR7	Acute graft *vs.* host disease, skin transplantation	Li et al. (2013), Dong et al. (2017)
Roxatidine	Inhibit CCR7 downstream MAPK signaling	Contact hypersensitivity	Lee et al. (2017)
Y27632	Block CCR7 downstream Rho signaling	Contact hypersensitivity	Nitschke et al. (2012)
Resolvin E1, U-75302	Change CCR7-stimulated metabolic reprogramming	Contact hypersensitivity	Sawada et al. (2015)
Anti-matrix metalloproteinase-9 Ab	Destroy CCR7-stimulated cytoskeleton rearrangement and adhesion	Rheumatoid arthritis	He et al. (2018)

### Immunotherapy Targeting CCR7 Directly

Preventive or therapeutic treatment of humanized CCR7 mice with anti-human CCR7 mAb 8H3-16A12 can restrain or relieve the rheumatoid arthritis mouse model effectually due to the weakened migration of DCs (Moschovakis et al., 2019). In the same way, in the low-risk setting of corneal transplantation, after the local injection of CCR7 blocking fusion protein, the DCs in dLNs were dramatically reduced, together with a significant elevation of graft survival rate, indicating a potential therapeutic strategy to clinical transplant rejection (Hos et al., 2016). Moreover, intervening in the regulatory network of CCR7 also shows great prospect for inflammatory disease remission. Cyclosporin A, triptolide, and curcumin were found to inhibit LPS-triggered upregulation of COX-2, PGE2, NF-κB, and AP-1 along with subsequent CCR7 expression, thus impairing the chemotaxis of DCs to CCL19/21 and motivating tolerance in diseases (Chen et al., 2004; Liu et al., 2007; Rahimi et al., 2021). Semaphorin 3E may regulate IRF4 to alter CCR7 levels and benefit allergic airway hyperresponsiveness (Movassagh et al., 2021). Moreover, the activation of PPARγ by selective PPARγ agonists, rosiglitazone, and ciglitazone impeded the CCR7 expression as well as the migratory properties of DCs, resulting in mitigated lung inflammation (Hammad et al., 2004; Fainaru et al., 2005). In psoriasis or rheumatoid arthritis, respectively, anti-TNF or FTY720 therapy downregulated CCR7 on DCs and then reduced DC aggregation in the lesional skin or dLNs, although the precise mechanism was still obfuscated (Bose et al., 2013; Han et al., 2015).

On the other hand, it is also well known that some tumor cells can damage the homing of DCs by suppressing their CCR7 expression to achieve immune escape. Therefore, reinforcing the expression of CCR7 in DCs may potentially be a versatile and potent therapy for resistant tumors (Mitchell et al., 2015; Nahas et al., 2018). For instance, sterol metabolism factors derived from human melanoma cell lines as well as colon, lung, and kidney carcinomas can inhibit CCR7 expression by triggering LXR-α activation. Using zaragozic acid, an inhibitor of the squalene synthase enzyme, to block cholesterol and oxysterol synthesis in tumors recovered CCR7 level and the migration ability of DCs, potentiating the antitumor effect of the single CD25-depleting monoclonal antibody treatment (Villablanca et al., 2010). Profiting from the amplified expression of CCR7, DCs pretreated with thyroid hormone triiodothyronine or allogeneic melanoma-derived cell lysate can move to dLNs and stimulate antigen-specific cytotoxic T-cell responses more effectively after being injected into melanoma animal models (Gonzalez et al., 2014; Alamino et al., 2015). Besides, CCR7 overexpressed and antigen modificatory DCs also show greater migratory ability to dLNs and more valid anti-tumor properties in melanoma and lung cancer (Okada et al., 2005; Salem et al., 2021). Interestingly, researchers have wrapped codeliver antigen OVA and plasmid DNA encoding *Ccr7* (*Ccr7* pDNA) in micelles to construct a novel immune adjuvant targeting micelles (M-COSA). M-COSA/OVA/pDNA vaccination effectively targets DCs and increases DC migration, thereby alleviating melanoma growth, providing good ideas for new vaccine design (Xiqin Yang et al., 2018).

In addition, since iDCs with a low migration rate can also swarm to dLNs and induce immunological tolerance, tolerogenic DCs with semi-mature phenotype are promising therapeutic tools for immune diseases (Guindi et al., 2018). iDC processed by a PKC inhibitor possessed augmented expression of CCR7 and migration rate to lymph nodes, which will activate functional Tregs greatly and show satisfactory tolerance-inducing effect for rheumatoid arthritis and primary Sjögren’s syndrome (Adnan et al., 2016). On acute graft *vs.* host disease in an allergenic bone marrow transposed mouse model, the injection of CCR7-modified iDCs extended the survival time of recipient mice due to the enhancive iDC migration (Li et al., 2013). Analogously, researchers exploited Ad-*Ccr7* and *RelB*-siRNA to simultaneously overexpress CCR7 and inhibit RelB (a member of the NF-κB family that can motivate iDC maturation) expression in iDCs. Encouragingly, the migration rate of iDCs was strikingly accelerated, the immune tolerance by upregulated Tregs was strengthened, and the survival rate was also elevated after iDC transfusion in a mouse skin transplantation model, further implicating the potential value of DCs for therapeutic intervention (Dong et al., 2017).

Given that GPCRs are excellent drug targets, some drugs have already been marketed for chemokine receptors, such as maraviroc for CCR5 and plerixafor for CXCR4 (Insel et al., 2019; Jorgensen et al., 2021; Zhang et al., 2021). However, there are no drugs targeting CCR7 in the market up to now. Cosalane and ICT13069 were reported as CCR7 inhibitors, though their possible role in DC migration is still unclear (Ahmed et al., 2017; Hull-Ryde et al., 2018). Structural studies of CCR7 are conducive to the discovery of possible binding sites and further specific antagonists or agonists of it. This way, navarixin (SCH-527123 or MK-7123) and Cmp2105 were found to stabilize CCR7 in an inactive state through interactions with its intracellular side (Jaeger et al., 2019; Saha and Shukla, 2020). In addition, navarixin is undergoing several clinical trials currently, including psoriasis, allergen-induced asthma, and chronic obstructive pulmonary disease, indicating its potential to benefit DC-related clinical immunotherapy (Salem et al., 2021).

### Immunotherapy Targeting the Downstream of CCR7

CCR7 alone is not sufficient for successful DC migration. It requires a range of downstream signal elements activated by it to accomplish this function, among which MEK/MAPK, Rho/cofilin, and PI3K/AKT pathways seem to be best known (Rodriguez-Fernandez and Criado-Garcia, 2020; Liu et al., 2021). Thus, targeting these signal elements may also be a good strategy for regulating DC migration-related diseases.

The MAPK family covers extracellular signal-regulated kinase (ERK), p38, and c-Jun N-terminal kinase (JNK), all of which are activated after CCR7 stimulation. Several MAPK inhibitors, such as U0126 for ERK, SB203580 for p38, and SP600125 for JNK, were found to hinder DC migration (Riol-Blanco et al., 2005). Roxatidine, used to treat gastric and duodenal ulcers, can suppress the accumulation of DCs in dLNs in chemical allergen-induced contact hypersensitivity, which may also be by the mechanism of attenuated MAPK signal (Lee et al., 2017). Moreover, inhibition of MEK, the upstream molecules of ERK, by U0126 and PD184352, can effectively ease contact hypersensitivity responses and rheumatoid arthritis (Thiel et al., 2007; Miyagaki et al., 2011). However, whether they function through DCs directly remains to be investigated. Many other MEK inhibitors, such as GSK1120212, PD0325901, and AZD6244, have been approved by the FDA or are being evaluated in clinical trials for oncotherapy (Han et al., 2021); it will be intriguing to investigate whether these inhibitors also participate in the regulation of inflammatory disease triggered by DC migration.

CCR7 also regulates the Rho signaling pathway, which will further mediate Pyk2 and ROCK components, thereby controlling the downstream targets cofilin and MLC, resulting in actin polymerization or contraction and cytoskeletal rearrangements (Xu et al., 2014). In ovalbumin (OVA)-induced *Gab1* knockout mice models of asthma, CCR7 expression was hardly changed while Pyk2 activation and DC migration were obviously restrained, thus considerably attenuating allergic inflammation. The inhibition of Pyk2 by PF-431396 distinctly reduced the migration of DCs induced by CCL19. Further studies found that Gab1 was able to interact with CCR7, Rho, and p115-RhoGEF. However, the assembly of Rho with p115-RhoGEF rather than CCR7 can be disrupted in the absence of Gab1(Zhang et al., 2016). On the other hand, the blockade of ROCK by Y27632 profoundly reduced the speed of DC migration from the skin to dLNs during both steady state and tissue inflammation, which may be beneficial to contact hypersensitivity-induced ear skin inflammation (Nitschke et al., 2012). Interestingly, the oral JAK inhibitor ruxolitinib was found to impair DC migration *via* off-target inhibition of ROCK but not to alter the expression of CCR7, adding further pieces to the puzzle to the mechanism of its immune-suppressive effects (Rudolph et al., 2016).

Previous studies have stated that ERK/p38/JNK and Rho/cofilin mainly affect the chemotaxis or migration speed, while PI3K/AKT works in the antiapoptotic signaling in DCs (Riol-Blanco et al., 2005). However, several recent investigations raised objections to this opinion. CCR7 stimulation gives rise to a significant activation of PI3K/AKT. Inhibition of AKT with AKT-IV, its canonical activator PI3K with wortmannin, or its downstream transcriptional factor hypoxia-inducible factor-1α (HIF-1α) with 2-methoxyestradiol were all shown to mightily block DC migration, emphasizing the importance of PI3K/AKT in DC migration. Researchers further found that HIF-1α can promote glycolysis to regulate DC migration, while long non-coding RNA lnc-Dpf3 can inhibit HIF-1α activation *via* the HRE motif, therefore inhibiting CCR7-triggered DC migration and alleviating contact hypersensitivity (Liu et al., 2019). Another study also showed that HIF-1α-dependent glycolytic metabolism, whose induction or long-term maintenance was mediated by the activation of AKT, mTOR, and TBK, is essential for cell morphology, CCR7 oligomerization, and DC migration (Cabrera-Ortega et al., 2017; Guak et al., 2018).

In a word, CCR7 ultimately functions through intracellular G-protein-dependent signal transducers, including MEK/MAPK, Rho/cofilin, and PI3K/AKT, to activate DC migration. These signal elements can work synergistically or separately, and some of these, in turn, can even alter the expression of CCR7 (Hogstad et al., 2018). It is pressing and pregnant to discover more compounds regulating these signaling elements, thus with great connotation for CCR7-related immunological diseases.

### Immunotherapy Targeting the CCR7-Activated Metabolic Program

Current models have noted that accompanying metabolic activities also have a role in supporting DC migration. Unbiased metabolomic profiling and metabolic pathway enrichment analysis in mDCs reveal that CCL19/21 stimulation will alter glycolysis metabolic pathways and induce the accumulation of glucose metabolic intermediates, which may be related to the AKT/HIF-1α signaling in part. This change fuels mitochondrial respiration by supplying carbon sources and expediting the oxidation of NADH to NAD^+^ by lactate dehydrogenase A in the final step of aerobic glycolysis, thus maintaining F-actin polarization and promoting DC migration (Liu et al., 2019). Deprivation of glucose will impair the elongated shape, motility, and migration of DCs (Guak et al., 2018). In addition, not only glycolytic inhibitor 2-deoxyglucose but also the mitochondrial ATP synthase inhibitor oligomycin and lactate dehydrogenase A inhibitor oxamate can abrogate CCR7-triggered DC migration both *in vitro* and *in vivo* (Everts et al., 2014; Liu et al., 2019); however, whether these inhibitors affect CCR7-related disease progression should also be examined.

In contrast, lipid accumulation such as cholesterol and oxysterol in DCs seems to inhibit DC migration, which may be associated with the activation of the PPARs and LXRα partially (Villablanca et al., 2010; Plebanek et al., 2020). Mice treated with resolvin E1, a lipid mediator derived from ω3 polyunsaturated fatty acids, exhibited a significantly reduced number of cutaneous migrated DCs in dLNs and suppressed T-cell induction and ear swelling response in the sensitization phase of contact hypersensitivity. Mechanistically, BLT1 induced actin filament reorganization and fortified DC motility, while resolvin E1 can block this signaling efficaciously by binding to and inhibiting BLT1. Hence, BLT1 antagonist U-75302 exerted similar effects to round DCs in shape, impairing CCR7-induced DC migration and skin inflammation (Sawada et al., 2015). Additionally, short-chain fatty acids butyrate and propionate can downregulate CCL19 or 5-(tetradecyloxy)-2-furoic acid and inhibit glycolysis in DCs, prompting their potential role in DC migration and CCR7-related diseases (Nastasi et al., 2015; Nguyen-Phuong et al., 2021).

### Immunotherapy Targeting CCR7-Stimulated Cytoskeleton Rearrangement

With the occurrence of a plethora of physiological and biochemical reactions aroused by the CCR7-CCL19/21 axis, the intracellular actin or cytoskeleton distribution shifts from a balanced status to a specific region, causing the appearance of the pseudopodia. Through the extension or shortening of the pseudopodia as well as the changes in stiffness, adhesion, or digestion properties, DCs move toward the target site along the chemokine gradient (Leithner et al., 2016; Joosten et al., 2018). Changing these intracellular activities is also a scope for more strategies that regulate DC migration-related diseases.

The CCR7 axis triggered changes in the cytoskeleton, especially the actin polymerization and retrograde flow at the front and the actinomyosin contraction at the rear, which is the presupposition for efficient chemotaxis of DCs (Moreau et al., 2018; Stankevicins et al., 2020). It depends on two main actin pools: the Cdc42-Arp2/3-dependent actin pool presented at the front, which limits iDC migration but promotes antigen capture, and the RhoA-mDia1-dependent actin pool located at the rear, which is used in both mDCs and iDCs for forward locomotion. It was found that inhibiting Arp2/3 with CK666 or Cdc42 with ML141 in iDCs decreased the accumulation of F-actin at their front but did not increase their maturation, hinting a great strategy to accelerate iDC migration and fortify tolerance. In sharp contrast, the formin inhibitor SMIFH2 and the RhoA inhibitor C3 convertase impaired the fast and persistent migration mode along CCL21 gradients both *in vitro* and *in vivo*; it is worth testing the potential effects of these inhibitors in DC migration-related diseases (Vargas et al., 2016). Consistently, knockout of mDia1 also suppressed the directional migration of DCs to CCR7 ligand and benefited delayed hypersensitivity response (Tanizaki et al., 2010). On the other hand, calcium efflux from lysosomes by the ionic channel TRPML1 promoted the chemotaxis of mDCs to dLNs, while constitutive calcium release from the endoplasmic reticulum by IP3 receptors channels was identified as required for iDC migration, both of which may function by upholding myosin IIA activity (Solanes et al., 2015; Bretou et al., 2017). Interestingly, due to the full activation of TRPML1 in mDCs, treatment of iDCs but not mDCs with TRPML1 activator MLSA1 was sufficient to increase iDC migration speed to the values reached by mDCs but did not modify the surface expression of costimulatory molecules, which may also be a resultful way to arouse immune tolerance (Bretou et al., 2017). In addition, cholesterol accumulation in the lysosomal compartment correlated with the chronic loss of TRPML1 activity, which may also affect CCR7 signaling and the trafficking of DCs partly (Shen et al., 2012; Li et al., 2016).

Except for the chemotactic migration of DCs, cytoskeleton rearrangement also regulates DCs stiffness as well as adhesion, further functioning in interstitial migration or transendothelial migration. Low doses of latrunculin A, which depolymerized actin filaments, did not induce obvious changes in the overall actin cytoskeleton but prevented nuclear passage through constrictions. It was found that perinuclear Arp2/3-driven actin nucleation disrupted nuclear lamina and increased deformability, allowing DCs to migrate through narrow gaps rapidly and efficiently (Thiam et al., 2016). Moreover, deficient of small GTPase Cdc42 or its specific guanine nucleotide exchange factor DOCK8 did not affect the 2-dimensional surface migration but impaired DCs to crawl within 3-dimensional fibrillar networks or to transmigrate through the SCS floor due to the temporal and spatial dysregulation of leading-edge protrusions (Lammermann et al., 2009; Harada et al., 2012). Surprisingly, *Nlrp10* ^−/−^ mice had a profound defect in adaptive immunity in many autoimmune disease models including asthma, experimental autoimmune encephalomyelitis, and airway inflammation (Eisenbarth et al., 2012). *Nlrp10* deletion caused the mutation of *Dock8*, resulting in impaired cytoskeleton dynamics and DC migration (Krishnaswamy et al., 2015). The adhesion between DCs and epithelial cells (ECs) can be mediated by intercellular selectin and its ligands. DCs can cross the ECs by combining P-selectin glycoprotein ligand-1 with E-selectin or P-selectin expressed in vascular ECs, thus acting significantly in the progression of atherosclerosis (Ye et al., 2019). Vav1, a guanine nucleotide exchange factor for Rho family GTPases and cytoskeletal-reorganization effector, was critical for DC binding to fibronectin and regulated the distribution but not the formation of podosomes. *Vav1* ^−/−^ DCs had increased the rates of migration *in vivo* due to the inhibition of adhesion and integrin-mediated signaling (Spurrell et al., 2009). In multiple sclerosis models, EZH2 disrupted the binding of talin to F-actin and, thereby, promoted the turnover of adhesion structures by interacting with Vav1 to promote the methylation of talin1. Disrupting the interactions between EZH2 and Vav1 formed frequent, enlarged focal adhesions that were connected to stress fibers in DCs, impairing the transendothelial migration and restricting multiple sclerosis progression (Gunawan et al., 2015). In addition, matrix metalloproteinase-9 can degrade collagen IV and destroy the extracellular matrix and basement membrane, thus promoting the transport of DCs. Based on this, anti-matrix metalloproteinase-9 Ab was found to alleviate rheumatoid arthritis by impeding DCs trafficking (He et al., 2018).

## Conclusion and Perspective

CCR7, together with its ligands, CCL19 and CCL21, hinges in facilitating the accurate migration of DCs to dLNs. Inappropriate expression of CCR7 may cause DC migration disorders, thus touching off many immune diseases. To this end, modifying the CCR7 system may be beneficial to halt disease progression. Despite our increasing understanding of the regulatory network to CCR7 and the potential CCR7-based immunotherapy, many fundamental questions are still unanswered and follow-up studies in more detail are still required.

It is well known that DCs can serve the immune system by antigen uptake, maturation, migration, cytokine secretion, and T-cell activation, and the overlapping mechanism and progressive relationship of these functions are currently elusive. Similarly, despite the awareness of many key signal transducers and intracellular activities involved in the CCR7 system, their interconnections remain complex and fuzzy. Furthermore, DC migration goes far beyond CCR7-mediated move from peripheral tissues to dLNs. The trafficking of other DC subgroups (such as pre-DCs and pDCs through HEV) has yet to be determined; how DCs coordinate the CCR7 axis to achieve these processes is also a point worthy of attention. In addition, the marketed specific agonist or antagonist for CCR7 even other drugs that targeted CCR7-related activities are scarce currently. Most of the strategies described before have been carried out in animal models, and it remains to be checked whether these discoveries are equally valid for humans. Drugs that affect the CCR7 system are warranted to further screening and clinical trials. However, it is still worth watching about the adverse effects of CCR7-related drugs as CCR7 is also significant for upholding immunity homeostasis. This way, drugs that selectively change part of the signaling cascades are warranted, and DC vaccines may also be a promising option.
